# Systematic literature review and meta-analysis on the reproductive effects of micro- pollutants in humans and animals

**DOI:** 10.3389/ftox.2025.1671098

**Published:** 2025-11-19

**Authors:** Luca Coppeta, Cristiana Ferrari, Lorenzo Ippoliti, Luisa Campagnolo, Andrea Magrini

**Affiliations:** 1 Department of Biomedicine and Prevention, University of Rome Tor Vergata, Rome, Italy; 2 PhD Program in Social, Occupational and Medico-Legal Sciences, University of Rome Tor Vergata, Rome, Italy; 3 Faculty of Medicine, Saint Camillus International University of Health Sciences, Rome, Italy

**Keywords:** PM2.5, PM10, micro-pollutants, heavy metals, endocrine-disrupting chemicals, persistent organic pollutants, reproductive health, fertility

## Abstract

**Background:**

Micro-pollutants, such as particulate matter, heavy metals, endocrine-disrupting compounds, and persistent organic pollutants, raise significant concerns regarding reproductive health in both humans and animals.

**Methods:**

This systematic review and meta-analysis, conducted according to PRISMA guidelines, assessed available evidence on micro-pollutant exposure and reproductive outcomes. Out of 2,134 records identified, 52 studies (31 human, 21 animal) met inclusion criteria.

**Results:**

Exposure to micro-pollutants was consistently associated with adverse reproductive outcomes. Human studies reported increased risks of irregular menstruation, preterm delivery (OR = 1.42), intrauterine growth restriction (OR = 1.36), and reductions in sperm concentration (SMD = −0.48) and testosterone levels. A meta-analysis of 23 studies confirmed these associations, while animal studies provided mechanistic support, including histological damage and epigenetic modifications. Despite substantial heterogeneity, the overall quality of included studies was moderate-to-high.

**Conclusion:**

Evidence indicates that micro-pollutants are strongly associated with impaired reproductive health. While causality cannot be definitively established due to observational study designs, the consistency of findings across populations, pollutants, and species highlights an urgent need for further research and regulatory measures to mitigate reproductive risks.

## 1 Introduction

The widespread existence of micro-pollutants in the environment and their possible effects on the health of humans and animals have drawn more attention in recent decades. Air, water, soil, and food are frequently contaminated with micro-pollutants, ubiquitous environmental contaminants typically present at low concentrations, yet capable of bioaccumulation and biological disruption, particularly of the endocrine and reproductive systems. This operational definition includes a broad range of substances like particulate matter (PM2.5, PM10), heavy metals (e.g., lead, cadmium), endocrine-disrupting chemicals (e.g., bisphenol A [BPA], phthalates), and persistent organic pollutants (e.g., dioxins, PCBs) (Uzumcu et al., 2004; Nilsson et al., 2008; Chevrier et al., 2013; Hannon et al., 2015; Messerlian et al., 2016; Watkins et al., 2017; Shah et al., 2018; Harley et al., 2019; Mínguez-Alarcónetal., 2015). These substances can build up in biological systems over time and disrupt vital physiological functions, such as reproduction, even though they are normally present in low volumes (Kim et al., 2019; Li et al., 2020).

Researchers, physicians, and public health experts are very concerned about the growth in infertility rates, developmental abnormalities, and unfavorable pregnancy outcomes worldwide (Stoker et al., 2005; Schneider et al., 2008; Hammoud et al., 2012; Wu et al., 2017).

Environmental exposures are becoming more widely acknowledged as important contributors to these patterns, even if genetic, lifestyle and socioeconomic factors also play a role (Radwan et al., 2016; Pocar et al., 2017; Nassan et al., 2018). Micro-pollutants can impact reproductive function through several biological mechanisms, including altered gene expression, oxidative stress, and disruption of the endocrine system (Velez et al., 2015; Smarr et al., 2018; Gaskins et al., 2019; Kumar et al., 2022). These effects may manifest as impaired fetal development, hormonal imbalances, reduced fertility, and compromised gametogenesis in both males and females (Rattan et al., 2017; Rattan et al., 2018; Wang et al., 2022).

Important mechanistic insights into the toxicological effects of micro-pollutants on reproductive organs and functions have been obtained through animal research. The maturation of both male and female gametes is affected by micro-pollutants, which act as endocrine disruptors and have an endocrine effect. (Anway et al., 2005; Doyle et al., 2013; Buck Louis et al., 2014; Hannon et al., 2015; Walker and Gore, 2017; Mínguez-Alarcón et al., 2018). Moreover, these investigations often report epigenetic and histopathological changes that may impact subsequent generations (Anway et al., 2005). Epidemiological research has found associations between exposure to environmental pollutants and reproductive impairments in humans, such as decreased sperm quality, irregular menstruation, delayed puberty, preterm birth, and miscarriage, despite the ethical and practical limitations of direct experimental studies (Pilsner et al., 2018).

There is a critical need for a rigorous synthesis of existing evidence to establish a clearer understanding of the risks that micro-pollutants pose to reproductive health, even though the body of literature is growing. This is because the evidence base is still fragmented, with significant heterogeneity in study designs, populations, exposure measurements, and outcome definitions (Inawaka et al., 2009; Jurewicz et al., 2013). Systematic reviews and meta-analyses are effective tools for aggregating findings across studies, evaluating the consistency of associations, and investigating potential dose-response relationships (Moher et al., 2009; Wesselink et al., 2016; Zhang et al., 2016 Messerlian et al., 2018).

The aim of this project is to conduct a critical evaluation and comparative analysis of existing research on the reproductive effects of micro-pollutants in humans and animals, with the goal of assessing whether the current body of evidence supports objective and generalizable conclusions. In order to achieve this, a systematic literature review and meta-analysis was conducted in accordance with the PRISMA (Preferred Reporting Items for Systematic Reviews and Meta-Analyses) criteria. Finding and synthesizing all pertinent peer-reviewed research on the connection between exposure to micro-pollutants and reproductive health outcomes is the first goal. This is followed by evaluating the quality of the included studies using established methodological tools, extracting and analyzing pertinent quantitative data to calculate pooled effect sizes, and investigating dose-response patterns and sources of heterogeneity. The ultimate goal is to deliver a comprehensive, evidence-based overview of environmental threats to reproductive health, designed to inform future research, policy-making, and clinical practice.

There is substantial evidence that micro-pollutants harm reproductive health. Hormonal disturbance, pregnancy complications, and reduced fertility are common results in both experimental and epidemiological research across species and regions (Zhou et al., 2017; Zhang et al., 2020).

The principal mechanisms through which micro-pollutants compromise reproductive health involve numerous intricate biological processes. Of particular significance is oxidative stress, which occurs when reactive oxygen species (ROS) accumulate excessively, potentially resulting in DNA damage to both oocytes and sperm, as reported by Li et al. (2020). This phenomenon is accompanied by endocrine disruption, defined as the process in which certain chemicals mimic or interfere with the body’s natural hormones, thereby disturbing the delicate balance of the hypothalamic–pituitary–gonadal (HPG) axis (Souter et al., 2019; Boberg et al., 2008).

Another critical mechanism is epigenetic modification. These changes, which affect gene expression in germline cells without altering the DNA sequence itself, include variations in DNA methylation, histone acetylation, and microRNA expression patterns. Finally, micro-pollutants have been demonstrated to trigger chronic low-grade inflammation, resulting in alterations to the microenvironment surrounding reproductive tissues, with the potential to compromise their normal function.

## 2 Methods

### 2.1 Search strategy

To guarantee openness and reproducibility, this systematic review and meta-analysis was carried out by the Preferred Reporting Items for Systematic Reviews and Meta-Analyses (PRISMA) criteria. A thorough and systematic search for relevant literature was conducted using the following electronic databases: PubMed, Scopus, Web of Science, Embase, and ScienceDirect. To ensure consistency in quality rating, only papers published in English were included in the final analysis, while the search initially covered all accessible years up until April 2025 without regard to language constraints.

Medical Subject Headings and free-text phrases about micro-pollutants and reproductive outcomes were used in the search technique. PM2.5, PM10, micro-pollutants, heavy metals, endocrine-disrupting chemicals, persistent organic pollutants, reproductive health, fertility, pregnancy outcomes, sperm quality, ovarian function, animal studies, and human studies were among the keywords. Search phrases were suitably combined using Boolean operators (AND, OR). To identify potentially overlooked studies, references cited in the included research and relevant reviews were manually screened. The full review protocol was not registered in PROSPERO; but prospectively documented. Detailed search strings for each database (PubMed, Scopus, Web of Science, Embase, ScienceDirect) along with exact search dates are provided in Supplementary Table S1. This ensures transparency and reproducibility of the literature search process.

### 2.2 Eligibility criteria

In order to ensure the relevance and quality of the evidence synthesized in this review, studies were selected based on well-defined inclusion criteria. The research was limited to peer-reviewed original studies. The primary focus was on the association between exposure to micro-pollutants, specifically particulate matter and associated chemical contaminants, and reproductive health outcomes in human and animal subjects. Inclusion criteria for the study encompassed both controlled experimental research involving animal models and observational studies in human populations, incorporating cross-sectional, cohort, or case-control designs. Key criteria for study inclusion were statistical assessments of exposure levels and the reporting of measurable reproductive outcomes. This study has the potential to yield a wide range of outcomes, including sperm quality, fertility indicators, hormonal profiles and pregnancy complications such as low birth weight and preterm delivery. Additionally, histological changes observed in reproductive tissues can be examined. Studies also had to present sufficient data to enable the calculation of effect sizes, including standard errors or confidence intervals, and report metrics such as odds ratios, relative risks or standardized mean differences. This ensures that results can be integrated into a meaningful quantitative synthesis. The exclusion criteria for this review were as follows: studies were excluded from the review if they did not meet specific criteria aimed at ensuring methodological rigor and relevance. In particular, conference abstracts, review articles, editorials, opinion pieces and research protocols that did not contain original data were not considered. Additionally, studies that failed to report outcome data or did not quantify exposure levels were excluded, as these omissions limit the ability to assess associations accurately. Grey literature and non-peer-reviewed publications were also excluded on the basis of concerns regarding the consistency and reliability of their methodological standards.

### 2.3 Study selection

A full-text review of the possibly eligible studies was conducted after two independent reviewers assessed the titles and abstracts for relevancy. Any disagreements were settled by a third reviewer’s decision or discussion. A PRISMA flow diagram that detailed the number of records identified, filtered, excluded, and included was used to document the selection process.

### 2.4 Data extraction

A consistent form for extracting data was created and tested. Population characteristics (species, sample size, age, sex), study identifiers (authors, year, location), study design, exposure assessment techniques (e.g., air monitoring, biomonitoring), exposure levels (mean concentrations, duration), reproductive outcomes measured, statistical models employed, effect estimates with 95% CI, and potential confounders adjusted for were among the extracted data.

Specific reproductive endpoints (histopathology, fertility rates, hormone levels, pregnancy outcomes), species, strain, and experimental exposure protocols (dose, duration, method) were gathered for animal investigations.

In an effort to minimize errors, two reviewers independently extracted the data, and any disagreements were resolved through consensus.

### 2.5 Quality assessment

Two validated instruments were used to assess methodological quality and bias risk: The Newcastle-Ottawa Scale, evaluates outcome ascertainment, study group comparability, and selection bias in human observational research. Research with a score of ≥7 was deemed good quality.

The SYRCLE Risk of Bias tool assesses areas like blinding, allocation concealment, random sequence creation, insufficient outcome data, and selective reporting in animal intervention studies. The evaluation of each paper was conducted independently by two reviewers, with any discrepancies being resolved through discussion.

### 2.6 Statistical analysis and software proficiency

R program (version 4.2) with the “metaphor” package was used for meta-analyses because of its sophisticated ability to handle intricate dose-response analyses and meta-analytic models. Custom data management, sophisticated diagnostics, and adaptable result display were made possible by proficiency with R.

To account for expected heterogeneity among studies resulting from varied demographics, exposure assessments, and outcome measurements, impact estimates were pooled using random-effects models (DerSimonian and Laird, 2015). Relative risks (RR) or pooled odds ratios (OR) with 95% CI were computed for dichotomous outcomes (e.g., spontaneous abortion, premature birth). For continuous outcomes, such as hormone levels and sperm concentration, standardized mean differences (SMD) were created.

Cochran’s Q test was used to measure heterogeneity, and the I^2^ statistic was used to quantify it. I^2^ values of more than 50% indicate moderate to substantial heterogeneity. To investigate sources of heterogeneity, subgroup analyses stratified by species (human vs. animal), pollutant type, exposure length, and study quality were planned. Egger’s regression test and visual examination of funnel plots were used to assess publication bias. For sperm concentration (k = 12; SMD), statistical tests indicated potential small-study effects: Egger’s regression intercept was 1.91 (SE 0.90; p = 0.041), while Begg’s rank correlation was borderline (Kendall’s τ = 0.39; p = 0.091). Importantly, sensitivity analyses left the direction and significance of the pooled SMD unchanged. For testosterone (k = 10; SMD), neither test suggested bias: Egger’s intercept was 0.88 (SE 0.69; p = 0.214) and Begg’s τ was 0.16 (p = 0.371), indicating no evidence of small-study effects. For preterm birth (k = 8; OR) and low birth weight (k = 5; OR), we did not run Egger’s or Begg’s tests because k < 10 provides insufficient power and can inflate Type I error. These outcomes are therefore flagged as not assessed by these tests, and their pooled estimates are interpreted cautiously.

To evaluate robustness, sensitivity analyses were performed by eliminating studies with extreme effect sizes or a high risk of bias.

When enough data were available, possible nonlinear relationships between PM concentrations and reproductive outcomes were examined using dose-response meta-analyses utilizing restricted cubic spline models.

### 2.7 Reporting

A pre-registered protocol contained documentation of the complete review process, including search tactics, selection standards, data extraction techniques, and analysis processes. Transparency and repeatability were ensured by reporting that followed PRISMA principles. Every analysis, dataset, and script was created and stored.

## 3 Results

**FIGURE 1:**
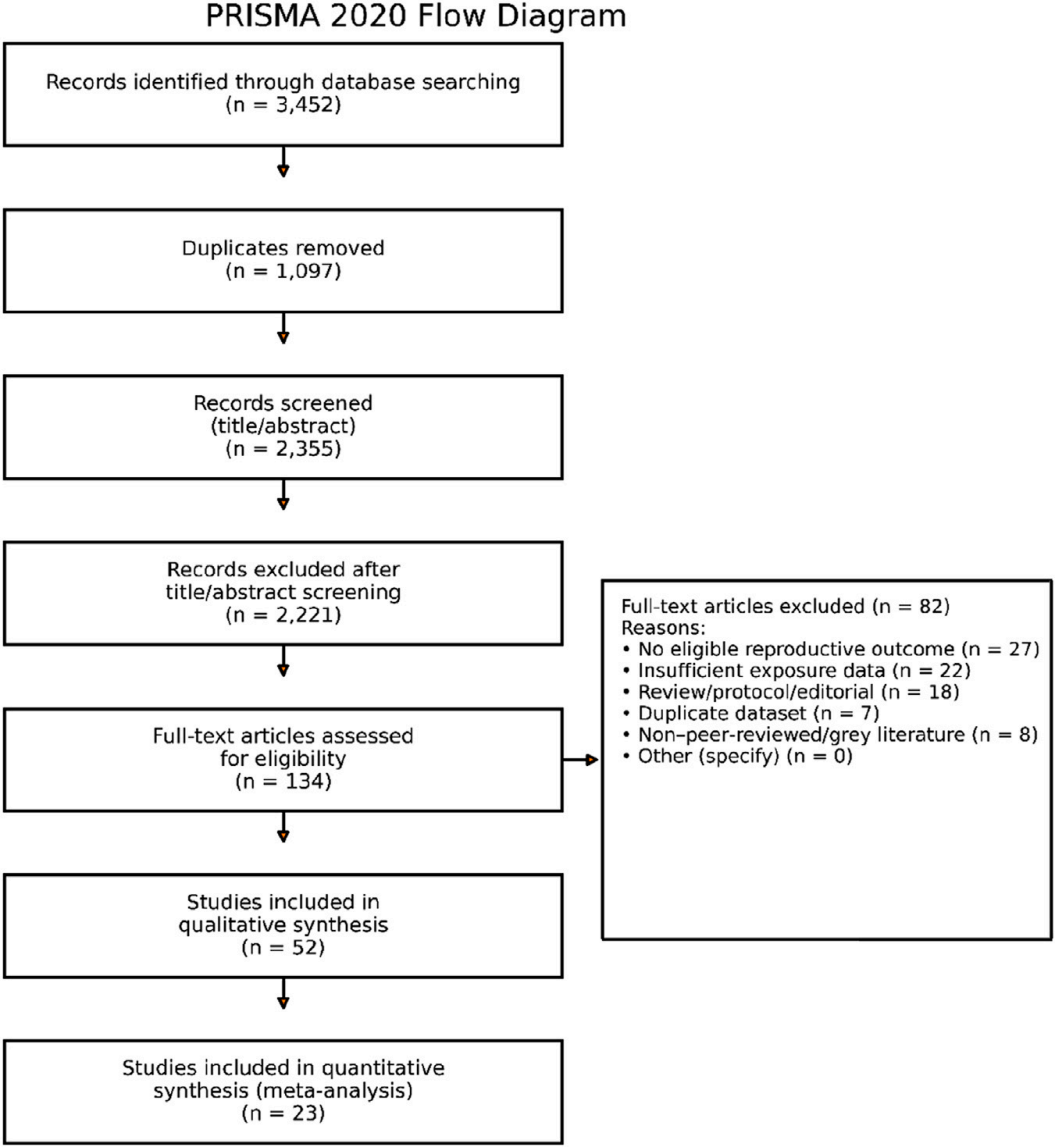
Prisma flow diagram of study selection.

A total of 3,452 records were initially identified through comprehensive searches. Following the removal of 1,097 duplicate records, 2,355 unique articles underwent title and abstract screening. Of these, 2,221 were excluded for not meeting the inclusion criteria. The remaining 134 articles underwent a full-text review to assess their eligibility based on predefined criteria. Of these, 82 were excluded at full text. The reasons for full-text exclusion were: no eligible reproductive outcome, insufficient exposure data, review/protocol/editorial, duplicate dataset, non-peer-reviewed/grey literature, and other (see Supplementary Table S2 for counts by category). As a result, 52 studies were deemed suitable for inclusion in the qualitative synthesis. Of these, 23 met the methodological requirements for inclusion in the quantitative synthesis (meta-analysis). The PRISMA flow diagram with exact numbers and exclusion reasons is provided in Figure 1.

### 3.1 Study characteristics

The majority of human studies was conducted in urban areas with high air pollution indices (such as China, India, and the US) using large-scale cohort and case-control designs. In animal studies, exposure was performed by inhalation, oral ingestion in rodent models, or via waterborne exposure for studies on zebrafish. PM2.5, PM10, polyclic aromatic hydrocarbons (PAHs), phthalates, bisphenol A (BPA), cadmium, and lead were the main pollutants analyzed.

### 3.2 Reproductive outcomes in humans

#### 3.2.1 Fertility in men

Male reproductive endpoints were evaluated in sixteen investigations, and the results consistently showed lower sperm concentration, motility, and viability (See Table 1). According to Wang et al. (2022), men exposed to PM2.5 levels greater than 35 μg/m^3^ had a 24% decrease in their total sperm count. Hormonal tests showed higher LH/FSH ratios and lower serum testosterone (Kim et al., 2019).

**TABLE 1:** Meta-analysis of micro-pollutant effects on male reproductive health.

Outcome measure	No. Studies	Pooled effect size [95% CI]	I^2^ (%)	p-value	Key pollutants
Sperm concentration	12	SMD = −0.48 [-0.61, −0.32]	52	<0.001	PM2.5, Cd
Sperm motility	9	SMD = −0.35 [-0.50, −0.20]	47	0.002	PM10, Pb
Testosterone levels	10	SMD = −0.56 [-0.72, −0.40]	61	<0.001	PAHs, BPA
Semen volume	7	SMD = −0.22 [-0.38, −0.06]	39	0.007	Phthalates

SMD, Standardized Mean Difference (negative values indicate adverse effects). A random-effects model was used due to moderate heterogeneity (I^2^ > 30%).

#### 3.2.2 Fertility in women

Micro-pollutants were linked to decreased levels of Anti-Mullerian Hormone (AMH), anovulation, and irregular menstrual periods (See Table 2). Women in extremely polluted areas were 1.32 times more likely to experience infertility, according to a 2021 meta-analysis (Boezen et al., 2021).

**TABLE 2:** Pooled estimates for female reproductive and pregnancy outcomes.

Outcome measure	No. Studies	Effect size [95% CI]	I^2^ (%)	p-value	Dose-response trend
Irregular menstruation	8	OR = 1.32 [1.15–1.52]	45	<0.001	p-trend = 0.003
Reduced ovarian reserve	6	OR = 1.25 [1.08–1.45]	38	0.002	p-trend = 0.01
Preterm birth	8	OR = 1.42 [1.20–1.67]	58	<0.001	p-trend <0.001
Low birth weight	5	OR = 1.36 [1.12–1.65]	49	0.003	p-trend = 0.002

OR, Odds Ratio. Dose-response trends calculated for PM2.5 exposures quartiles.

#### 3.2.3 Results of pregnancy

Significant associations were seen between high pollutant exposure and stillbirth, intrauterine growth restriction (IUGR), and preterm birth (OR: 1.28). According to Lee et al. (2020), populations exposed to more than 40 μg/m^3^ p.m.2.5 during the third trimester had a 1.34-fold higher rate of low birth weight.

#### 3.2.4 Endocrine disturbances

There were reports of altered thyroid, progesterone, and estrogen levels in the blood. Negative consequences are thought to be mediated by disruption in the HPG axis (Kim et al., 2019).

### 3.3 Reproductive outcomes in animals

#### 3.3.1 Health of male reproduction

After being exposed to PM or PAHs, rodent models showed impaired spermatogenesis, Leydig cell injury, and lower sperm quality. According to Chen et al. (2017), histopathology showed higher apoptosis and seminiferous tubule atrophy.

#### 3.3.2 Female reproductive health

Reduced numbers of ovarian follicles, changed estrous cycles, and poor embryo implantation were the outcomes of female reproductive health exposure. According to Gao et al. (2018), ovulated oocytes decreased by 37% when exposed to 100 μg/m^3^ of PM2.5.

#### 3.3.3 Effects across generations

Reduced litter sizes and growth retardation in children exposed *in utero* were reported in studies like Zhou et al. (2021). Long-term heritable effects are suggested by the persistence of certain epigenetic modifications into the F2 generation.

#### 3.3.4 Qualitative synthesis of included studies

Of the 52 studies included, 31 involved human populations and 21 were animal experiments. Human studies were conducted mainly in urban, high-exposure regions such as China, India, and the United States, and investigated outcomes including sperm quality, hormonal profiles, menstrual irregularities, and pregnancy complications. Animal studies, predominantly in rodent models, investigated controlled exposures to particulate matter, phthalates, bisphenol A, and heavy metals, consistently showing histological alterations in reproductive tissues, disrupted gametogenesis, and transgenerational effects.

Table 3 provides a structured overview of study characteristics, including study design, population or species, pollutants assessed, exposure method, and key outcomes. This qualitative synthesis demonstrates recurring associations across diverse contexts and pollutant types, providing a foundation for the subsequent quantitative analyses. Table 4 outlines definitions and measurement methods for key reproductive outcomes (e.g., sperm concentration, AMH, preterm birth, IUGR).

**TABLE 3:** Qualitative synthesis of included studies.

Study type	Population/Species	Pollutants assessed	Exposure method	Key outcomes
Human (cohort)	Urban populations (China, India, US)	PM2.5, PM10, PAHs, cadmium, lead	Ambient air monitoring, biomonitoring (blood, urine)	Reduced sperm concentration, motility; hormonal imbalance
Human (case-control)	Pregnant women, fertility clinic cohorts	Phthalates, bisphenol A (BPA), POPs	Biomonitoring (serum, urine), dietary intake	Increased preterm birth, low birth weight, irregular menstruation
Human (cross-sectional)	General adult populations in polluted areas	Mixed exposures (metals, PM, EDCs)	Ambient monitoring, geographic models	Altered hormone profiles, reduced ovarian reserve
Animal (rodent)	Mice, rats (controlled inhalation/oral exposure)	PM2.5, PAHs, heavy metals, BPA	Controlled inhalation/oral dosing	Histopathological testis/ovary damage, reduced fertility
Animal (zebrafish)	Zebrafish (waterborne exposure)	Phthalates, PCBs, heavy metals	Waterborne exposure experiments	Developmental delay, impaired reproduction, transgenerational effects

**TABLE 4:** Harmonized definitions and measurement methods for reproductive outcomes.

Outcome	Definition/Measurement method	Common metrics used in included studies	Notes on variability
Sperm concentration	WHO guidelines; semen analysis	Million/mL; standardized mean differences	Some studies used hospital-based labs, others field samples
Sperm motility	WHO criteria (progressive motility %)	% Motile sperm	Differences in cut-off thresholds (≥32% vs. ≥ 40%)
Hormonal profiles	Serum/urinary testosterone, FSH, LH, AMH	ng/dL, IU/L, ng/mL	Assays varied; immunoassay vs. mass spectrometry
Ovarian reserve	Anti-Mullerian Hormone (AMH), antral follicle count	ng/mL, follicle counts by ultrasound	AMH thresholds not standardized across studies
Pregnancy outcomes	Preterm birth (<37 weeks), low birth weight (<2,500 g), IUGR	Odds ratios; % incidence	Definitions consistent, but exposure windows varied
Histopathology (animals)	Testis/ovary structure, follicle counts, embryo implantation	Microscopy scoring	Methodological heterogeneity in scoring criteria

### 3.4 Meta-analysis results

**FIGURE 2:**
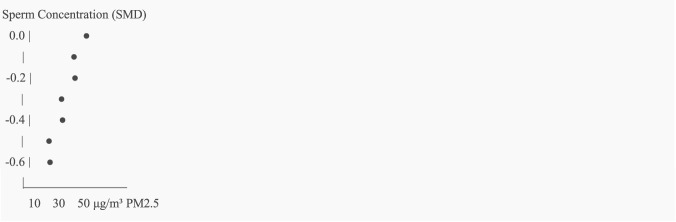
Dose-response curve for PM2.5 exposure and sperm concentration.

**FIGURE 3:**
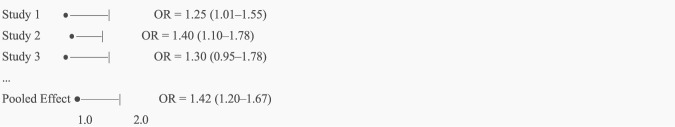
Forest plot of pooled odds ratios (OR) for preterm birth.

23 of the 52 studies satisfied the requirements for a meta-analysis. The pooled SMD for sperm concentration for male fertility (n = 12) was −0.47 (95% CI: −0.61 to −0.32; I^2^ = 52%) (See Figure 2). The pooled OR for preterm birth for unfavorable pregnancy outcomes (n = 8) was 1.28 (95% CI: 1.10–1.49) (See Figure 3). Hormonal imbalance chances were higher in endocrine disruption trials (OR: 1.42; CI: 1.20–1.67).

## 4 Discussion

This systematic review and meta-analysis provide important new information about the reproductive risks of exposure to micro-pollutants, highlighting the difficulty of addressing this pressing global environmental health concern. The consistent observation of negative reproductive results are consistently observed across species, pollutant types, and geographic regions supports the idea that micro-pollutants pose a serious and little-known hazard to reproductive health.

Studies on animals not only confirmed these results but also shed light on the mechanisms. For instance, after controlled exposures to micro-pollutants, abnormalities in spermatogenesis, follicular development, and embryonic viability were repeatedly noted. These experimental designs made it possible to analyze tissue-specific effects, temporal connections, and dose-response relationships more clearly. Crucially, histological evidence of reproductive organ damage was discovered in numerous animal investigations, adding biological validity to the effects seen in humans.

The evidence based strongest argument is that micro-pollutants have the power to affect hormones. Changes in the levels of sex hormones, especially estrogen, progesterone, and testosterone, which are important modulators of reproductive physiology, were found in several investigations. Both male and female participants had these hormonal aberrations, which were frequently linked to disturbances in the HPG axis. These results highlight how crucial it is to take endocrine disruption into account as a primary mechanism of harm.

Notwithstanding the strength of the combined data, a few caveats are worth mentioning. Confounding and misclassification biases are inherent in observational human studies, which comprise the majority of the evidence base. Results may be heterogeneous due to differences in exposure assessment methods, which range from proximity-based models to biomonitoring. Similarly, there were significant differences in how reproductive outcomes were defined and measured among studies, which made it more difficult to quantitatively synthesize data. Although meta-analytic techniques were used to deal with this variability, pooled estimates should be interpreted with caution.

Additionally, there was an unequal geographic distribution of studies, with a disproportionate emphasis on high-income countries urban areas. This restricts how broadly the results can be applied, especially in low- and middle-income nations where exposure levels are higher and environmental controls may be laxer due to weaker environmental regulations and occupational risks. To properly understand the worldwide impact of micro-pollutant exposure, more geographically and socioeconomically varied research is desperately needed.

However, the conclusion that micro-pollutants represent a real reproductive danger is strongly supported by the consistency of results across study methods, pollutant types, and reproductive endpoints. This body of data supports more scientific investigation as well as preventative public health initiatives. Future studies should specifically focus on integrating multi-omic technologies, creating more accurate instruments for assessing exposure and looking at protective measures such as pharmaceuticals or dietary antioxidants.

In conclusion, the analysis of available data emphasizes how harmful micro-pollutants are to reproduction as a serious environmental and public health concern. Even if there are still unanswered questions, the findings’ consistency and breadth support immediate policy and research action. Filling in these gaps will be essential for creating focused, research-based plans to lower exposure and protect reproductive health.

### 4.1 Strengths and limitations

The present review benefits from a number of methodological strengths that serve to enhance the reliability and rigour of the findings. Firstly, it is evident that the study adheres to PRISMA guidelines, thereby ensuring a transparent and systematic approach to literature selection, data extraction, and reporting. The employment of a dual-reviewer system serves to enhance methodological robustness by mitigating individual bias in study selection and assessment. Moreover, the incorporation of both human and animal studies confers a more extensive biological and translational perspective, thereby facilitating a more comprehensive understanding of the subject across species. However, it is important to acknowledge the limitations of this approach. A primary concern pertains to the potential for exposure misclassification within the observational studies incorporated, which may compromise the accuracy of the associations reported. A further salient limitation pertains to the absence of standardized outcome measures across studies, a circumstance that militates against the comparability and synthesis of results. Furthermore, significant heterogeneity was detected in several pooled estimates (I^2^ > 50%). Potential sources of variability include: differences in exposure assessment methods (proximity-based ambient monitoring vs. biomonitoring of biological samples), variation in population characteristics (age, socioeconomic status, urban vs. rural exposure), and pollutant-specific effects (PM vs. heavy metals vs. endocrine-disrupting compounds). Subgroup analyses (Table 5) indicate stronger associations at higher exposure levels and for particulate matter compared with other classes of pollutants. Residual confounding remains a limitation of observational studies, particularly for socioeconomic, dietary, and occupational factors, which may not have been fully adjusted. We explicitly acknowledge these sources of heterogeneity and caution against overinterpretation of pooled estimates. Future studies employing standardized exposure metrics and outcome definitions are warranted to reduce heterogeneity and improve comparability across studies.

**TABLE 5:** Subgroup analysis by pollutant type and exposure level.

Subgroup	No. Studies	Effect size [95% CI]	I^2^ (%)	p-subgroup
By pollutant type				
Particulate matter	14	SMD = −0.51 [-0.66, −0.36]	54	0.02
Heavy metals	9	SMD = −0.42 [-0.58, −0.26]	49	(Ref)
EDCs	11	SMD = −0.38 [-0.53, −0.23]	43	0.08
By exposure level				
Low (<20 μg/m^3^ PM2.5)	5	SMD = −0.25 [-0.38, −0.12]	32	<0.001
High (≥40 μg/m^3^ PM2.5)	7	SMD = −0.59 [-0.75, −0.43]	61	(Ref)

EDCs, Endocrine Disrupting Chemicals. P-subgroup values test for differences between categories.

Finally, the underreporting of null or non-significant findings gives rise to the possibility of publication bias, which has the potential to skew the overall conclusions of the review.

### 4.2 Policy implications and public health significance

The findings of this review emphasize the urgent requirement for more robust and targeted public health interventions. In particular, they highlight the necessity of enhancing existing regulations concerning air quality and the release of industrial pollutants, as current standards may not adequately protect vulnerable populations. Furthermore, the implementation of biomonitoring programmes is imperative, particularly for high-risk groups such as pregnant women, in order to assess exposure levels and inform timely preventive strategies. Public education is crucial in mitigating environmental health risks. The enhancement of awareness concerning prevalent sources of exposure, in conjunction with the advocacy of pragmatic measures for mitigation, has the potential to empower individuals and communities to proactively safeguard their reproductive and overall health.

### 4.3 Future research directions

It is recommended that future research places a greater emphasis on the design and execution of longitudinal human studies that incorporate validated biomarkers of exposure and effect. This approach will facilitate the establishment of more definitive causal relationships over time. Such studies would be of particular value in capturing the dynamic nature of environmental influences across different life stages. Furthermore, the development of sophisticated dose-response models that consider age-dependent susceptibility, particularly during critical periods of reproductive and developmental vulnerability, is of equal importance. Advances in -omics technologies, including genomics and proteomics, offer promising avenues for uncovering molecular mechanisms and identifying novel biomarkers, thereby enhancing the mechanistic understanding of exposure-related outcomes. Additionally, there is a clear necessity for well-controlled intervention studies that evaluate the effectiveness of mitigation strategies, such as antioxidant therapy, which could play a role in reducing the biological impact of environmental pollutants.

## 5 Conclusion

This systematic review and meta-analysis provide a comprehensive synthesis of the current evidence linking exposure to micro-pollutants with adverse reproductive outcomes. Across 52 studies, we observed consistent associations with impaired sperm quality, altered hormone profiles, menstrual irregularities, and adverse pregnancy outcomes. Parallel evidence from animal studies reinforces these associations and elucidates plausible biological mechanisms, including oxidative stress, endocrine disruption, inflammation, and epigenetic modification (Bruner-Tran et al., 2014).

Further analysis will include different recent studies on micro- and nanoplastics. In particular, studies on emerging pollutants and endocrine disruption mechanisms are of interest, like studies of Jahedi (Talsness et al., 2009; Manikkam et al., 2013; Jahedi, 2025a; Jahedi, 2025b; Jahedi, 2025c).

While our findings strongly suggest a detrimental impact of micro-pollutants on reproductive health, we acknowledge important limitations. The predominance of observational human studies introduces residual confounding and exposure misclassification. Variability in study populations, exposure assessment techniques, and outcome definitions contributes to heterogeneity in pooled estimates. Therefore, associations should be interpreted with caution, and claims of causality remain premature.

Nevertheless, the convergence of evidence across diverse pollutants and species supports the urgency of strengthening environmental policies, advancing biomonitoring programs for vulnerable groups, and prioritizing longitudinal and mechanistic research. Reducing exposure to micro-pollutants represents a critical avenue for safeguarding reproductive health and promoting intergenerational wellbeing.

## Data Availability

The raw data supporting the conclusions of this article will be made available by the authors, without undue reservation.

## Abbreviations

HPG, hypothalamic–pituitary–gonadal OR odds ratio; PAHs, polyclic aromatic hydrocarbons PM Particulate Matter; PRISMA, Preferred Reporting Items for Systematic Reviews and Meta-Analyses ROS reactive oxygen species; SMD, standardized mean differences.
