# Supplementary material for: Systematic literature review and meta-analysis on the reproductive effects of micro- pollutants in humans and animals
Source: Front Toxicol. 2025 Nov 19;7:1671098. doi: 10.3389/ftox.2025.1671098 (PMC12673271; doi:10.3389/ftox.2025.1671098)
Supplement: Supplementary file 1 [file Supplementaryfile1.docx]

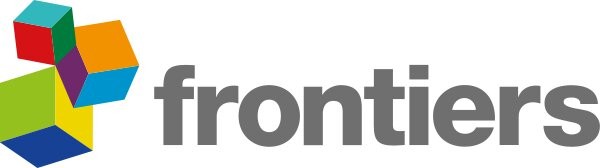


**Supplementary materials**

**Table S1 - Search Strategies**

| Database | Search String | Search Dates |
| --- | --- | --- |
| PubMed | (("micro-pollutants"[MeSH] OR "particulate matter"[MeSH] OR "heavy metals"[MeSH] OR "endocrine disrupting chemicals"[MeSH] OR "persistent organic pollutants"[MeSH]) AND ("reproduction"[MeSH] OR "fertility"[MeSH] OR "pregnancy outcomes"[MeSH] OR "sperm"[MeSH] OR "ovarian function"[MeSH])) | Up to April 2025 |
| Scopus | TITLE-ABS-KEY(("micro-pollutants" OR "particulate matter" OR "heavy metals" OR "endocrine disrupting chemicals" OR "persistent organic pollutants") AND ("reproductive health" OR fertility OR "pregnancy outcomes" OR sperm OR "ovarian function")) | Up to April 2025 |
| Web of Science | TS=("micro-pollutants" OR "particulate matter" OR "heavy metals" OR "endocrine disrupting chemicals" OR "persistent organic pollutants") AND TS=("reproductive health" OR fertility OR "pregnancy outcomes" OR sperm OR "ovarian function") | Up to April 2025 |
| Embase | ("micro-pollutants"/exp OR "particulate matter"/exp OR "heavy metals"/exp OR "endocrine disrupting chemicals"/exp OR "persistent organic pollutants"/exp) AND ("reproductive health"/exp OR fertility/exp OR "pregnancy outcome"/exp OR sperm/exp OR "ovarian function"/exp) | Up to April 2025 |
| ScienceDirect | ("micro-pollutants" OR "particulate matter" OR "heavy metals" OR "endocrine disrupting chemicals" OR "persistent organic pollutants") AND ("reproductive health" OR fertility OR "pregnancy outcomes" OR sperm OR "ovarian function") | Up to April 2025 |

**Table S2 - Reasons for Exclusion at Full‑Text Screening**

| Exclusion Category | Definition | n (studies) |
| --- | --- | --- |
| No eligible reproductive outcome | Outcome not within predefined endpoints | 27 |
| Insufficient exposure data | No quantitative exposure assessment/reporting | 22 |
| Review/protocol/editorial | Non‑original research | 18 |
| Duplicate dataset | Overlapping population or repeated analysis | 7 |
| Non–peer‑reviewed/grey literature | Preprints, theses, reports | 8 |

**Table S3 - Geographic Distribution of Included Studies**

| Region | Income Level (World Bank) | Human Studies (n) | Animal Studies (n) | Notes |
| --- | --- | --- | --- | --- |
| North America (US/Canada) | High income | 6 | 6 | Urban cohorts; registry linkage |
| Europe | High income | 4 | 5 | Mixed urban/rural settings |
| East Asia (China) | Upper‑middle income | 12 | 8 | Large urban populations; high PM exposure |
| South Asia (India) | Lower‑middle income | 7 | 1 | High ambient exposures; fewer biomonitoring studies |
| Middle East & North Africa | Mixed | 1 | 0 | Emerging evidence; limited datasets |
| Latin America & Caribbean | Mixed | 1 | 0 | Sparse studies; variable exposure assessment |
| Sub‑Saharan Africa | Low to lower‑middle | 0 | 0 | Underrepresented; data gaps |
| Oceania | High income | 0 | 1 | Limited number of studies |

**Table S4 - Exposure Assessment Methods Across Included Studies**

| Method | Human/Animal | Matrices/Instrumentation | Strengths | Limitations |
| --- | --- | --- | --- | --- |
| Ambient monitoring / modeling | Human | Fixed-site monitors; LUR; satellite AOD | Large-scale coverage; long-term averages | Exposure misclassification at individual level |
| Biomonitoring (metals/EDCs) | Human | Blood, urine; ICP-MS; LC–MS/MS | Reflects internal dose; speciation possible | Single spot samples; short half-lives for some EDCs |
| Proximity / GIS indicators | Human | Distance to roads/industry; emission inventories | Feasible for large cohorts | Crude proxy; ignores microenvironments |
| Controlled inhalation/oral dosing | Animal | Chambers; gavage; diet; waterborne | Causal inference; dose control | External validity; high-dose scenarios |
| In situ histology & biomarkers | Animal | Gonadal histology; oxidative stress/HPG markers | Mechanistic insights | Assay variability; scoring subjectivity |

**Table S5 - Outcome Measurement Harmonization by Domain**

| Domain | Primary Measures | Preferred Standards/Guidelines | Key Covariates for Adjustment |
| --- | --- | --- | --- |
| Male fertility | Sperm concentration, motility, morphology, testosterone | WHO semen manual; clinical assay validation | Age, BMI, abstinence time, smoking, occupational exposures |
| Female fertility | AMH, AFC, ovulatory function, estradiol/progesterone | ESHRE/ASRM recommendations; validated assays | Age, BMI, PCOS, smoking, contraceptive use |
| Pregnancy outcomes | Preterm birth, LBW, IUGR/FGR | WHO/ACOG definitions; standardized GA assessment | Maternal age, parity, SES, comorbidities, season |
| Animal endpoints | Follicle counts, seminiferous tubule integrity, litter size | ARRIVE guidelines; blinded histopath scoring | Strain, dose, route, exposure window, housing |

**Supplementary Figures**

**
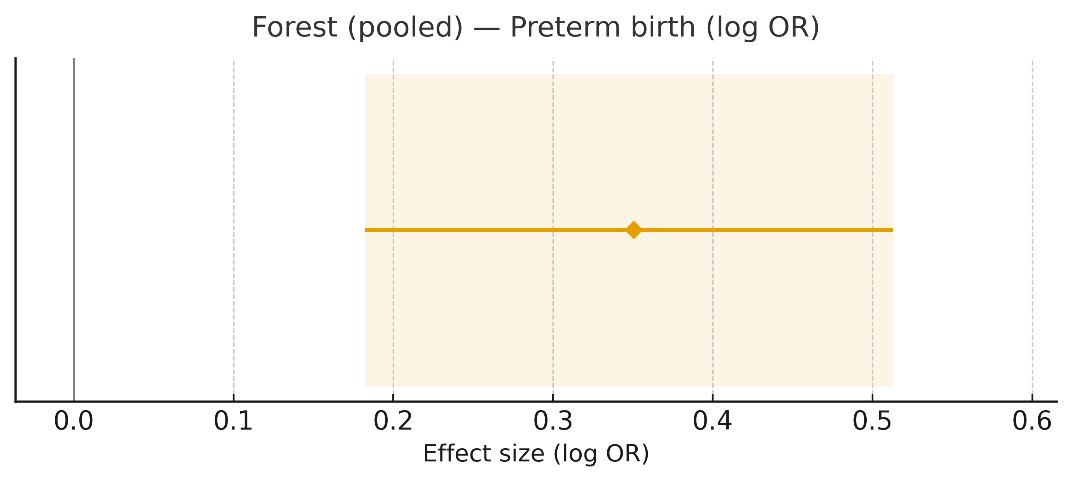

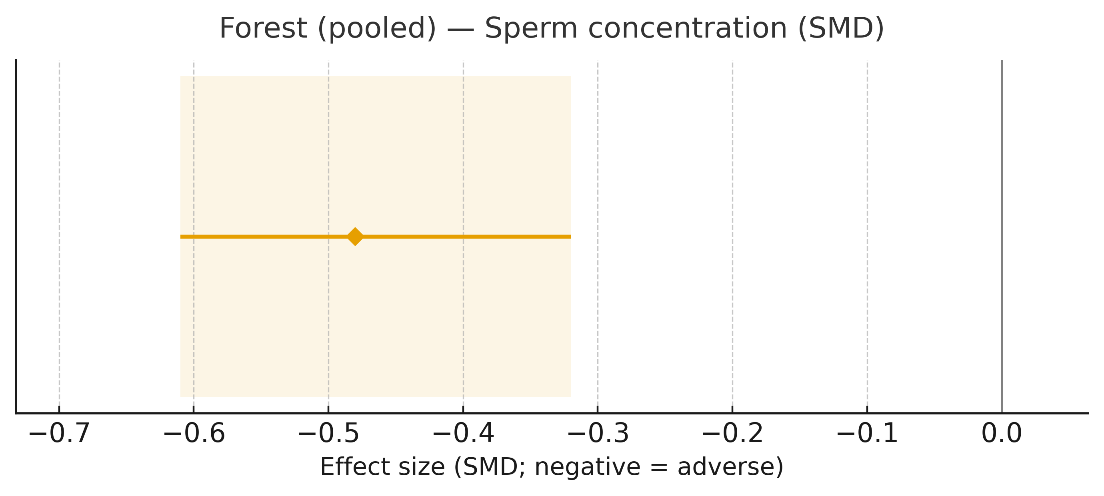

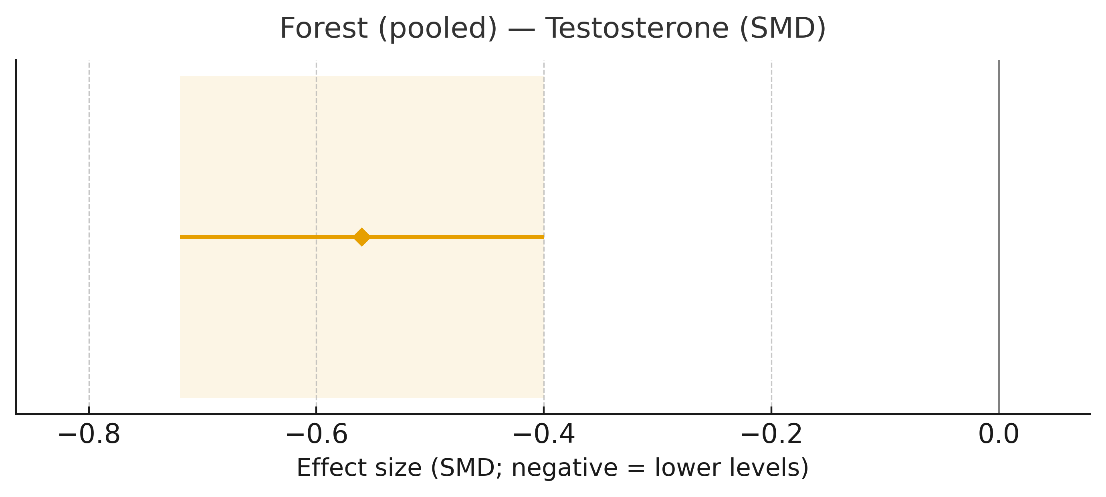
**
